# Effects of Task-Specific and Strength Training on Simulated Military Task Performance in Soldiers

**DOI:** 10.3390/ijerph17218000

**Published:** 2020-10-30

**Authors:** Tommi Ojanen, Keijo Häkkinen, Jaakko Hanhikoski, Heikki Kyröläinen

**Affiliations:** 1 Finnish Defence Research Agency, Finnish Defence Forces, P.O. Box 5, 04401 Järvenpää, Finland; 2 Biology of Physical Activity, Faculty of Sport and Health Sciences, University of Jyväskylä, P.O. Box 35, 40014 Jyväskylä, Finland; Keijo.Hakkinen@jyu.fi (K.H.); jaakkohanhikoski@gmail.com (J.H.); heikki.kyrolainen@jyu.fi (H.K.); 3 National Defence University, Finnish Defence Forces, P.O. Box 7, 00861 Helsinki, Finland

**Keywords:** neuromuscular performance, strength training, task-specific, occupational test, military, soldier

## Abstract

A soldier’s occupational physical task requirements are diverse and varied. However, the type of physical training that most effectively improves soldiers’ occupational task requirements has not been studied previously. The purpose of this study was to determine the important strength characteristics for soldiers during a repeated simulated military task course, and the type of training that may be effective to improve these abilities during a specialized military training period. Forty-two (*n* = 42) soldiers participated in the study. They were divided into three training groups; a soldier task-specific training group (TSG, *n* = 17), a strength training group (STG, *n* = 15), and a control group (CON, *n* = 10). Participants were measured before (PRE), middle (MID) and after (POST) the 12-week training intervention for strength performance and simulated military task test. Simulated military task performance improved significantly in TSG and STG between the PRE and MID measurements (from 9.4 to 15.7%). TSG and STG improved in various spilt times, especially in strength tasks; casualty drag (from 8.3 to 13.6%) and kettlebell carry (from 13.2 to 22.4%) between the PRE and MID measurements. The present study showed that both the training of TSG and STG were more effective than the training of CON (control group) in terms of improving the performance in the repeated simulated military task course. The present study showed that training of TSG was as effective as STG to improve repeated simulated military task course time. Therefore, an optimal training combination should include high-intensity simulated military task field training and strength training programmed with consideration of the military training phase and environmental possibilities.

## 1. Introduction

Common occupational physical tasks for soldiers can include patrolling long distances in different terrains, carrying and lifting objects with variable weight, and performing explosive movements in the battlefield [1]. All these requirements create complexity for constructing physical training programs for soldiers. In all, various factors including sex, age, physical training history, nutrition, recovery, and sleep, in addition to psychological, environmental, and social aspects can significantly influence physical training adaptations [2]. Moreover, optimizing a soldier’s performance in military environments is constantly challenged by external stress factors including carrying loads of various weights, sleep deprivation, extended physical activity, negative fluid and energy balance, and continuous readiness [3,4,5]. These stressors have been shown to cause disruptions in hormonal balance [6,7,8,9], leading to reduced physical and cognitive performance [3,10,11,12,13], prolonged recovery times [14], and increased susceptibility to infections [15]. Therefore, training load combined with these external stress factors can lead to compromised training adaptations, overreaching or even overtraining, in addition to increased musculoskeletal injury rates [16,17,18]. All these factors should be carefully considered when planning and implementing optimal physical training programs for soldiers.

To meet the occupational requirements, it is important to determine the best training programs and periodization models for a soldier. Decreased overall physical activity and fitness of recruits creates more demands for their initial training [19]. The increase in daily activity in garrisons can result in a sudden increase in recruits’ physical activity, which increases the risk of overtraining or injury [16,18,20,21]. Therefore, it is crucial to have a well-planned and periodized physical training program taking into account the initial physical performance level. In the past, most physical programs have concentrated on improving aerobic endurance of recruits using traditional periodization [2]. A more suitable approach may be the use of an undulating or a block periodization model with elements of aerobic and anaerobic conditioning and strength training [22,23,24,25,26,27]. In addition, due to strict timetables, physical training should be incorporated into the occupational training of a soldier, and the majority of training should be performed wearing a uniform and in field conditions [28]. This would help soldiers to train more occupational skills and to better adapt to different terrains: possibly decreasing injuries during training. Both the decline in physical fitness and the higher demands in occupational requirements of the recruits have led to a situation, where systematically designed, tailored, and supervised physical training that meets the occupational task requirements needs to be performed during the basic training [29]. While designing a training plan for soldiers, it is essential to evaluate the components emphasizing the occupational task requirements. The result of physical training programs relies on training frequency, intensity (velocity and load), and volume (repetitions and duration), which form the foundation of training [2]. To enhance and optimize training adaptations and to reduce overtraining and training-related injuries in training, it is crucial to plan and organize the training individually. [30] (p. 205). Nevertheless, it is critical that these emphasized training factors are well related to individual needs, and the initial fitness level of the trainee, and the training plan is well periodized. Additionally, variation in training stimulus is one of the most crucial components when considering the development of explosive or maximal strength and maximal aerobic capacity [30] (pp. 260–263 and 299–300). Daily military training can be too monotonous and may not enhance a soldier’s physical performance by the best possible way. Professional soldiers should have access to training programs, which first helps them to reach and then to maintain the required level of physical performance [31].

In the past, there have been studies regarding specific occupational task tests for soldiers [1,32,33]. These studies have shown that a soldier needs both aerobic and anaerobic endurance and muscle strength to fulfill occupational requirements. Mala et al. [32] observed that strength and power were strongly related to high-intensity military tasks with and without heavy load carriage. Pihlainen et al. [33] showed that the maximal countermovement jump (CMJ), 3000 m running time, skeletal muscle mass, and repeated push-ups explained about 60% of military simulation test time. In addition, in a study by Sporis et al. [26] anaerobic endurance and strength were found to be important factors in the soldiers’ performance. According to the current literature, it seems that there is no “gold standard” to measure soldiers’ occupational physical performance. As a consequence, the most effective way to train to improve these occupational requirements has not been studied sufficiently. Thus, the purpose of this study was to determine which physical abilities are important for soldiers during a repeated simulated military task course and which type of training (task specific, strength, or traditional military fitness training) would be useful and contribute to gains in these abilities during a specialized military training period.

## 2. Materials and Methods

### 2.1. Subjects

A training group of forty-two (*n* = 42) male soldiers (age ranging from 18 to 22 years) participated in the study and performed all the measurements during the 12-week study period. Their mean (±SD) age was 20 (±1) years, height 180 (±6) cm, body mass 72.9 (±9.3) kg, and body mass index 22.6 (±2.3) kg·m^−2^ (Table 1). The subjects were all from the same infantry company, and they were divided into three training groups by their platoon as follows: soldier task specific training group (TSG), strength training group (STG), and control group (CON). The present study was conducted according to the provisions of the Declaration of Helsinki and the ethical statement of the Ethical Committee, the University of Jyväskylä. The study was approved by the Finnish Defence Forces. All the recruits were informed of the experimental design, and the benefits and possible risks that could be associated with the study prior to signing an informed consent to voluntary participate in the study.

### 2.2. Procedures

**Figure 1:**
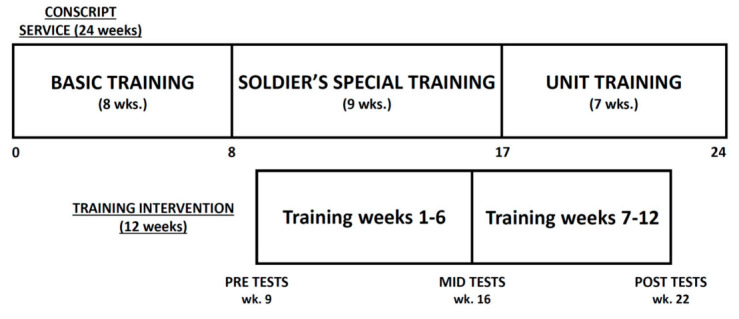
Timeline of the study.

The study was implemented during the latter part of the Finnish conscript service, after the eight-week basic training period (Figure 1). Physical performance tests and simulated military task performance were repeated three times during the training study, in the ninth week (PRE), sixteenth week (MID), and twenty-second week (POST) of their conscript service. The testing began in the morning with body composition measurements. After breakfast, the subjects performed muscular power, strength, and strength endurance tests. Finally, after lunch they performed the simulated military task performance test. Each training group completed their tests during the same day.

#### 2.2.1. Neuromuscular Performance

Maximal isometric bilateral force of the extensor muscles of the lower (MVClower) and upper (MVCupper) extremities was measured in a sitting position. The measurements were conducted using an electromechanical dynamometer [34] manufactured by the University of Jyväskylä (Jyväskylä, Finland). In the upper extremity test, the pushing bar was adjusted to the height of the shoulders and the distance of the seat was set to maintain an elbow angle of 90°. In the lower extremity test, the distance of the seat was set to maintain knee and hip angles of 107° and 110°, respectively. A countermovement jump (CMJ) was performed on a contact mat (Newtest, Oulu, Finland). Flight time between contacts was used to calculate the jumping height for each jump [35]. A six-second cycle ergometer test (Wattbike Ltd., Nottingham, UK) was used to measure maximal power of the lower extremities. The six-second test has a seated stationary start with a dominant leg initiating the first down-stroke. The test started following a five-second countdown followed by verbal command. The completion of the test was also indicated with another verbal command [36].

#### 2.2.2. Simulated Military Task Performance

**Figure 2:**
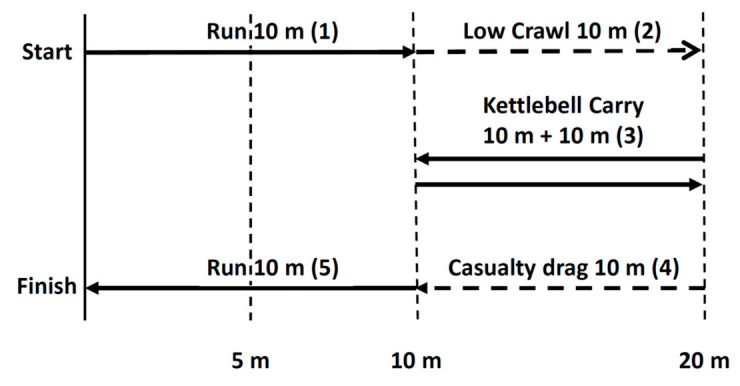
Outline of the simulated military task performance course. Different tasks are numbered in the order of performance (1–5).

The simulated military task performance consisted of typical army soldier tasks and maneuvers, such as sprints, crawling, carrying objects, and casualty evacuation. It was performed inside on an artificial turf with soldiers wearing typical combat gear, including the helmet (total extra weight of 22-kg). From the starting (lying) supine position, the soldiers performed a 10 m sprint, followed by a 10 m low crawl. After the low crawl, the subjects lifted, carried, and lowered two 16-kg kettlebells (CompactFit Ltd., Helsinki, Finland) twice for a distance of 10 m taking a supine position when lowering the kettlebells. This was followed by a 75-kg sandbag drag (Rogue Sandbag, Rogue Fitness Europe Ltd., Pori, Finland) for 10 m, followed by a sprint of 10 m. The total length of the track was 60 m (Figure 2).

The track was performed three times with a 60 s rest between the trials. Before the simulated military task performance, the subjects were individually familiarized with the track by a supervisor who also gave verbal instructions and encouraged the subjects during the test. The performance time was recorded by a stopwatch and video recorded for a later verification. The split times for different tasks were obtained offline from the video. Heart rate was recorded throughout the test by the Firstbeat team system (Firstbeat Technologies, Jyväskylä, Finland) and peak heart rate was determined from the data. Blood lactate was analyzed (Biosen c-line Sport, EKF Diagnostic, Madgeburg, Germany) from the fingertip (20 μL) five times (before, after each trial and 10 min after the final trial). The sensitivity for lactate analysis was 0.5 mmol/L and interassay coefficient of variation was 6.2%. Before and immediately after the simulated military task performance, the participants shot 10 rounds (Eko-Aims Ltd., Ylämylly, Finland) from a prone position with a similar army assault rifle replica (RK95, Finland) that they handle daily in their military training. The sum of ten shots was recorded for further analysis.

### 2.3. Training Protocols

Training programs were designed to be performed as a part of the recruits’ normal weekly physical training. During the 12-week training period, the total number of instructed physical training sessions performed by the recruits was 12 during the first six weeks and six sessions during the latter six weeks of the study. They also conducted their normal military training every day, which consisted of road marches, shooting exercises and other task specific exercises. Variation of the instructed physical training program was due to military field training (MFT), which was a part of their training program. During those weeks, the conscripts were not able to perform any extra physical training. All the groups participated in the instructed physical training, but the content of training varied between the groups. All of them performed 10 min active warm-up, which included body weight movements, active stretching, and running, before starting the actual exercise. The total length of one session was 60 min in all groups. The average daily RPE (Borg’s scale 6–20) varied during the training days as follows; TSG 10.7–13.4, STG 10.2–14.7, and CON 10.2–14.4 without significant differences between the groups. Training sessions of the TSG group included basic infantry-based exercise with full combat gear (27 kg), such as sprints, crawling, and casualty drag. Exercises were performed with anaerobic emphasis for 30–60 s. The STG group trained with the non-linear strength training program. The full-body program included squats, hamstring curls, pull and push exercises for the upper body, and different core exercises. The program began with low-load and high-repetition (40–60% of one-repetition maximum (1RM), 12–15 reps), continued to moderate-load and volume (70–85% of 1RM, 6–12 reps) culminating in high-load and low-volume training (85–100% of 1RM, 1–4 reps). CON performed normal Finnish Army military physical training only. A typical training session included circuit training with body weight, running with a constant pace, or playing different ball games. The training protocols for each group are presented in more detail in the previously published paper [37].

### 2.4. Statistical Analysis

The data for the present study was analyzed using commercial statistical software (IBM SPSS 24.0 Chicago, IL, USA). All data were checked for normality when calculating descriptive values for all variables. Conventional statistics were used to calculate means, 95% confidence intervals, and standard deviations (±SD). Effects sizes were calculated according to Cohen [38] (0.2 = small; 0.5 = medium; and 0.8 = large effect). Probability adjusted *t* tests were used for pairwise comparisons when appropriate. A general linear model, with repeated measures ANOVA with group as a fixed factor, was used to analyze the time × group interaction and the differences between the different measuring points. Associations between physical performance and military task test were performed using a Pearson correlation coefficient. Statistical significance for this investigation was set at *p* ≤ 0.05 (two-tailed).

## 3. Results

### 3.1. Neuromuscular Tests

Maximal isometric strength produced during the bilateral leg press increased significantly between the PRE and MID and PRE and POST measurements in TSG, but not in STG and CON. Maximal isometric strength during the bilateral bench press increased significantly in STG between the PRE and MID and PRE and POST measurements. In TSG, a significant increase occurred between the MID and POST measurements. No significant differences between the groups were observed in these maximal isometric forces.

In all groups significant increases occurred in 6 s maximal anaerobic power in the cycle ergometer test between PRE and POST (Table 2). TSG and STG also showed significant increases in power between the PRE and MID measurements, but not CON. In CMJ, a significant increase occurred in jump height between PRE and POST in TSG and between MID and POST in TSG. No significant differences were observed between the groups in the changes in CMJ.

### 3.2. Simulated Military Task Performance

#### 3.2.1. Total Time

**Figure 3:**
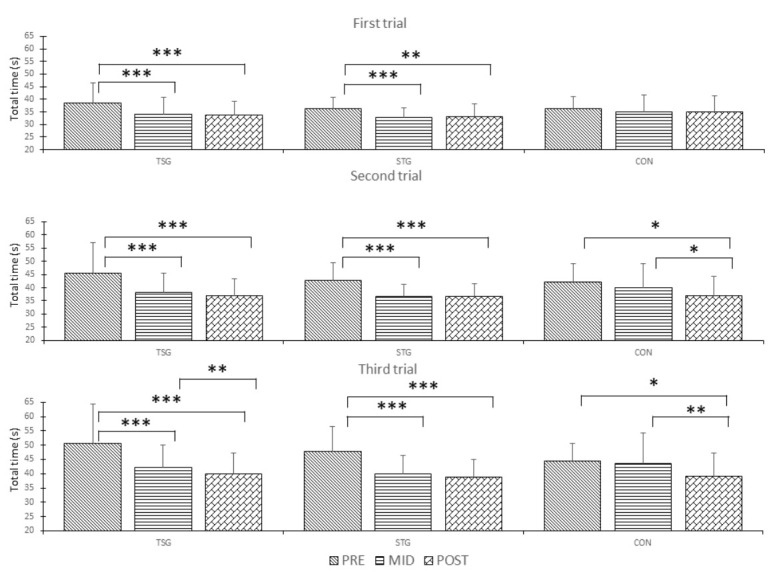
Total time (s) in the simulated military task course (first, second, and third trial). STG = strength training group; TSG = soldier task specific group; CON = control group; * = *p* < 0.05, ** = *p* < 0.01, *** = *p* < 0.001.

The total time of the first trial in the simulated military task performance improved in TSG and STG between the PRE and MID measurements by 11.1% (ES = 0.64) and 9.4% (ES = 0.91) and between the PRE and POST measurements by 11.4% (ES = 0.74) and 8.8% (ES = 0.72), respectively. No significant changes were observed in CON between the measurements in the first trial. In the second trial, the total time improved significantly in TSG and STG between the PRE and MID measurements by 14.6% and 13.1%. All groups showed significant improvements between the PRE and POST measurements (TSG: 17.0%, STG: 14.1%, and CON: 12.0%, ES = 1.35, 1.12, and 0.76, respectively). There was also a significant improvement in CON between the MID and POST measurements (6.3%, ES = 0.36). A similar trend was also observed in the third trial. Significant improvement was found between the PRE and MID measurements in TSG (14.6%, ES = 0.78) and STG (15.7%, ES = 1.05), but not in CON (2.1%, ES = 0.08). Between the PRE and POST measurements, a significant improvement occurred in all groups (TSG 19.2%, ES = 1.01, STG 18.2%, ES = 1.23, and CON 12.1%, ES = 0.77). TSG and CON showed a significant improvement between the MID and POST measurements (TSG 5.3%, ES = 0.32, and CON 9.6%, ES = 0.52; Figure 3).

#### 3.2.2. Five Meters Run from the Start

No significant changes were found in running time over the first 5 m during the first trial. (Table 3) During the second trial, TSG and CON significantly improved between the PRE and POST measurements. (Table 4) In the third trial, TSG and STG significantly improved between PRE and MID. CON improved significantly only between the PRE and POST measurements (Table 5).

#### 3.2.3. Ten Meters Crawl

TSG and STG significantly improved their time in the first trial between the PRE and MID and PRE and POST measurements. (Table 3) In the second trial, TSG and STG improved crawling time between PRE and MID and between PRE and POST. CON improved crawling time between the PRE and POST measurements. TSG and CON also improved their time between MID and POST (Table 4). In the 3rd run, TSG and STG improved crawling time between PRE and MID and between PRE and POST. CON improved time between PRE and POST. Between MID and POST, there was a significant improvement in TSG and CON (Table 5).

#### 3.2.4. Ten + Ten Meters Kettlebell Carry

TSG and STG improved carry time in the first trial between the PRE and MID measurements. TSG also improved its time between the PRE and POST measurements (Table 3). In the second trial, TSG and STG improved their carry time between PRE and MID and between PRE and POST. CON also improved its time between the PRE and POST measurements. STG and CON improved their time between the MID and POST measurements (Table 4). TSG and STG improved their carry time in the third trial between PRE and MID and PRE and POST. CON also improved its time between the PRE and POST measurements. STG and CON improved their time between the MID and POST measurements (Table 5).

#### 3.2.5. Mannequin Drag

In 75 kg mannequin drag, there were improvements in the first trial in TSG and STG between the PRE and MID and PRE and POST measurements. (Table 3) Similar results were also observed between the PRE and MID measurements in TSG and STG in the second trial (Table 4) and in the third trial (Table 5), and between PRE and POST in the second trial (Table 4) and in the third trial (Table 5). No significant changes in the mannequin drag were found in CON.

#### 3.2.6. Ten Meters Run

There were significant improvements during the first trail in TSG and STG between PRE and MID and PRE and POST (Table 3). Furthermore, during the second trial, there were significant improvements in TSG and STG between PRE and MID and PRE and POST. In addition, CON improved its time between PRE and POST (Table 4). In the third trial, TSG improved its time between PRE and MID and between PRE and POST. STG showed an improvement between PRE and POST (Table 5).

#### 3.2.7. Blood Lactate and Heart Rate

Blood lactate and heart rate increased throughout the simulated military task performance in all measurement points. The highest values in blood lactate were measured in the POST measurement in all groups (ranging from 15.08 to 16.90 mmol/L). Mean heart rate varied between 172 and 182 bpm during the simulated military task performance.

#### 3.2.8. Associations between Body Composition, Physical Tests, and Simulated Military Task Performance

Absolute individual changes in physical tests and times in the simulated military task performance correlated negatively and significantly in the PRE (*p* < 0.05; r = −0.366 to −0.659), MID (r = −0.352 to −0.789), and POST(r = −0.338 to −0.725) measurements. Furthermore, absolute individual changes in physical performance and individual changes in the simulated military task performance, correlated significantly between the PRE–MID measurements in isometric leg press force (second trial; r = −0.396, *p* = 0.009) and in maximal power (first trial; r = −0.348, *p* = 0.026). Similar correlations were found between the PRE–POST measurements in leg strength (second trial; r = −0.333, *p* = 0.031), maximal power (first trial; r = −0.429, *p* = 0.005), and CMJ (first trial; r = −0.451, *p* = 0.003). In the MID and POST measurements, no significant correlations between the changes in different tasks were found.

**Figure 4:**
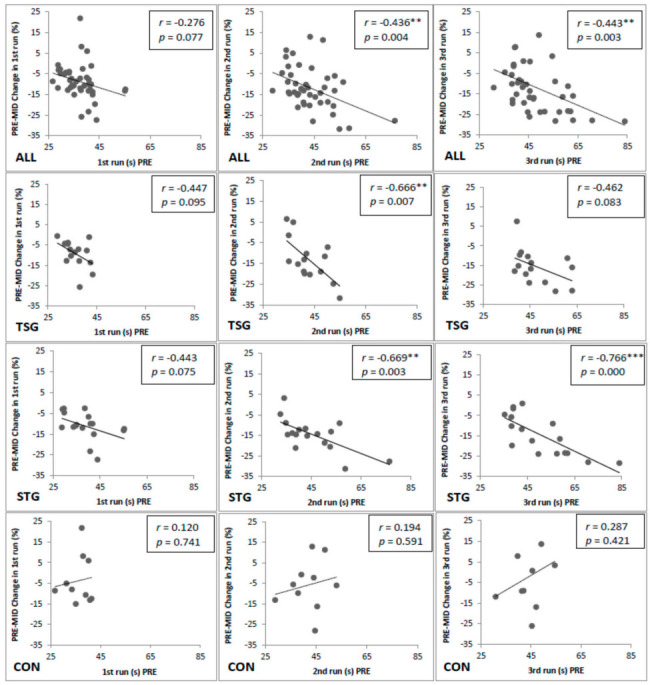
Correlations between absolute time in the 1st, 2nd, and 3rd run and the PRE–MID change (%) in time. ALL = all subjects; STG = strength training group; TSG = soldier task specific group; CON = control group; * = *p* < 0.05, ** = *p* < 0.01, *** = *p* < 0.001.

When looking at the relative change in time in the PRE–MID time points compared to absolute PRE times, there was significant negative correlations in STG (r = −0.669, *p* = 0.003) and TSG (r = −0.666, *p* = 0.007) in the second trial and in STG (r = −0.766, *p* = 0.000) in the third trial. In the CON group, respectively, no significant correlation was found (Figure 4).

## 4. Discussion

The main finding of the present study showed that STG and TSG improved performance in the simulated military task course more than CON during the first six weeks of the study period (PRE–MID). During the second six weeks of the study (MID–POST), the subjects were able to maintain physical fitness and simulated military task course performance despite the high amount of military field training and the decreased number of physical training sessions.

It is necessary for soldiers to have an appropriate physical performance level, before military field training (MFT) or deployment. As seen previously, MFT may have unfavorable effects on soldier’s physical performance and activity levels [16,20]. Hence, it is crucial to have an adequate recovery period after a long military training course or MFT to retrieve combat readiness [39]. Altogether, military training and deployments create a complicated environment for strength development due to the fact that endurance training is fully integrated into soldiers’ daily training. It has been shown that high (>3 times/week) endurance training frequency, especially with high training volumes, may have a negative influence on strength and, specifically, explosive performance during their concurrent training [40,41,42]. Daily military training may also have a similar effect on the development of different physical characteristics.

When considering the physical performance tests, no significant changes were found between the training groups in all measurements. In the six-second cycling test, maximal power increased in all groups, but STG and TSG improved most between the PRE and MID measurements. In the maximal strength test, only significant improvements were observed in leg press in the TSG group and in bench press in the STG group. In CMJ, small significant improvements were noticed in the TSG and STG groups. Although no differences between groups were observed in the present study, greater improvements took place in the TSG and STG groups in the strength and power tests compared to the CON group. In addition, the improvements were similar in the TSG and STG groups. Thus, it seems that task-specific training is as effective as strength training to improve soldiers’ military task specific performance. This finding is in line with a previous study done in the military environment [43], where no differences between the training groups were observed.

The total number of training sessions during the 12-week study period was 18. The first six weeks of the study was the actual training intervention period including 12 training sessions. Between the PRE and MID measurements, there was only one MFT, which lasted five days. During other weeks, the subjects had two to three training sessions as described earlier in the methods. The last six weeks of our study can be described as a maintenance period, because the subjects had four different MFTs during this period, lasting from four days to ten days. During this period the subjects had only six training sessions, zero to three times in a week. It has been shown earlier [44,45] that more than two sessions per week should be implemented to obtain improvements in strength and power performance in the military environment. In addition, the influence of military training [44,45] might have affected the outcome of the present study, especially, during the last six weeks.

Although no significant differences were found between TSG and STG in the simulated military task course, they both improved more than the CON group, especially, between the PRE and MID measurements. In addition, TSG seemed to have larger improvements in most parts of the course compared to STG, which may be explained by specific training with the same tasks as the actual simulated course in TSG [46]. It is also important to notice that the other groups had similar drills during the regular military training, but with lower volume and intensity. The results in our study support the findings by Harman et al. [43], where the training groups did high-intensity task-specific training as well. These improvements can also be explained by increased aerobic capacity, which could not be measured reliably in the present study. High-intensity interval training, performed by TSG, has been shown to lead to improvements in both aerobic and anaerobic performance [47]. Thus, when considering the results, it is possible that the task-specific training had more effect on improving both aerobic and anaerobic performance than strength-focused training. With regard to the improvement in the casualty drag, which involves moving a heavy load as fast as possible, both TSG and STG improved their performance significantly, but no significant changes were found in CON. This was probably due to the training of CON not including any maximal strength or high-power type of exercises. It has been shown in previous studies [29,32] that the ability to produce force and power is important in improving performance in these kinds of tasks. If the casualty drag would have been longer, most likely greater differences between TSG and STG compared to CON might have been observed. Furthermore, some indicators may demonstrate the specificity of the training, such as STG improvements in isometric bench press between the PRE and MID measurements while TSG did not, and TSG improved maximal isometric leg press force between PRE and MID while STG did not. This was most likely due to the fact that the main focus in the TSG group was in lower body training, when STG performed also upper body exercises. This finding should be taken into account when planning training programs in the future. Upper body maximal strength has been shown to be important for soldiers’ performance [48,49], and especially with field-based training, and must be taken into account when designing training programs. In addition, motivation has been shown to have a major impact to actual performance in these kinds of military task courses. Although all groups had high lactate values, CON seemed to have slightly lower concentrations compared to TSG and STG, but this might be because of their training, which did not include high intensity training.

With regard to the associations between different variables, there was a good correlation between the CMJ and six-second cycling power tests as compared to improved performance in the simulated military task course. This has also been found in previous studies when comparing power production and different task specific courses [32,50,51]. We also observed a significant correlation between time over the first five meters run and maximal upper body strength and push-up tests. Mala et al. [32] also found a similar association in their study. This can be explained by the type of task, which was performed starting in the prone position followed by standing up to run. This involves upper body extensors, which may be an important factor to perform the actual task [51]. The present study also showed that increased fat-free mass correlated significantly with the improvement in the simulated military task performance and with the increase in maximal isometric strength. These findings have been [32,51] shown to be important factors in evacuation tasks that involve high loads. When considering the PRE times and relative changes between the PRE and MID measurements, there was a significant correlation between the PRE time and magnitude of improvement in TSG and STG. Both task-specific and strength training programs were highly effective for the subjects who performed worse in the PRE measurements. Normal physical training did not have the same effect in CON.

In the present study, an endurance training group was planned to be included in the experimental design but because of a low number of subjects in this group, we had to drop it out of the study. The endurance group and a long duration endurance test would have provided more information on overall physical performance of the simulated military task performance. It should also be pointed out that the small number of the subjects were not fully familiar with all of our measurements prior to the study, because they had missed the familiarization session, but all the subjects were individually instructed in detail before the measurements. In addition, it should be remembered that all tasks in the simulated military test were the same as they performed throughout during their basic military training. In the future studies, it is important to include an endurance test in order to determine what physical abilities matter most in the simulated military task course.

The present study showed that both the task-specific and strength training programs were more effective than that of CON during the first six weeks in improving the performance in the repeated simulated military task course. It is important to have high-intensity training alongside with low-intensity military training to improve soldiers’ task specific performance. The present study showed that task-specific training is as effective as that of strength training to improve the repeated simulated military task course time. This is an important finding, because this kind of training can be carried out without a high amount of equipment and in large groups compared to gym-based strength training. An optimal combination could include high-intensity simulated military task field training and strength focused gym training depending on the military training phase and environmental possibilities. In addition, an intensive six-week training period during the specialized military training can improve physical and military occupational performance in previously trained soldiers. In future studies it is important to compare different training programs over a longer follow-up period. In addition, it seems that it is also possible to maintain these levels of physical and military occupational performance during a six-week intensive military field-training period with only a few physical training sessions.

## 5. Conclusions

In summary, daily physical training recommendations should be dependent on the conventional characters of military training, which includes low-intensity and high-volume endurance training with an additional load of 25–65 kg. For this reason, progressively advancing combined strength, power, and endurance training including some proportion of high intensity interval training or microtraining seems to induce superior adaptations in a soldier’s physical performance. In order to create more effective development in physical adaptations and performance, expanding attention should move towards more progressive and individualized physical training programs, which are split into phases that continuously improve performance. Therefore, an individualized approach to human performance optimization should be taken when trying to improve a soldier’s physical fitness and to reach better operational readiness.

## Figures and Tables

**Table 1:** Mean (±SD) age, height, body mass, and body mass index (BMI) in the study groups.

Variable	TSG (*n* = 17)	STG (*n* = 15)	CON (*n* = 10)
Age (years.)	20 (±1)	20 (±1)	20 (±1)
Height (cm)	180 (±7)	183 (±6)	177 (±4)
Body mass (kg)	73.2 (±9.8)	73.8 (±7.8)	71.1 (±11.0)
BMI (kg·m^−2^)	22.7 (±2.2)	22.1 (±1.7)	22.7 (±3.7)

TSG, task specific group; STG, strength training group; CON, control group; BMI, body mass index.

**Table 2:** Mean ± SD, 95% confidence interval (CI), and effect size values in the maximal neuromuscular tests in the PRE, MID, and POST measurement points.

		PRE		MID		POST		|Effect Size|		
	Group	Mean (±SD)	95% CI	Mean (±SD)	95% CI	Mean (±SD)	95% CI	1 vs. 2	2 vs. 3	1 vs. 3
CMJ (cm)	TSG	29 ± 6	26; 32	29 ± 5	26; 31	31 ± 4 *^,#^	28; 33	0.00	0.43	0.38
	STG	33 ± 6	29; 36	33 ± 5	30; 36	35 ± 4	32; 37	0.00	0.43	0.38
	CON	32 ± 6	28; 36	31 ± 7	26; 35	31 ± 6	27; 35	0.15	0.00	0.16
POWER (w)	TSG	1036 ± 140	961; 1110	1078 ± 138 *	1004; 1151	1094 ± 125 **	1027; 1160	0.30	0.12	0.43
	STG	1097 ± 98	1043; 1152	1140 ± 92 *	1090; 1192	1141 ± 103 *	1084; 1198	0.44	0.01	0.43
	CON	974 ± 122	886; 1061	1007 ± 144	904; 1110	1027 ± 147 *	922; 1132	0.24	0.13	0.38
UPPER BODY STRENGTH (kg)	TSG	78 ± 14	71; 86	77 ± 13	70; 84	80 ± 12 ^##^	74; 87	0.07	0.23	0.15
	STG	79 ± 14	72; 87	83 ± 14 *	75; 90	84 ± 14 **	77; 92	0.28	0.06	0.35
	CON	75 ± 14	65; 85	75 ± 15	65; 86	77 ± 16	65; 88	0.00	0.17	0.18
LOWER BODY STRENGTH (kg)	TSG	236 ± 40	214; 257	252 ± 39 *	231; 273	255 ± 50 **	239; 284	0.40	0.07	0.41
	STG	229 ± 49	202; 256	242 ± 39	221; 264	244 ± 39	223; 266	0.29	0.05	0.33
	CON	224 ± 50	189; 260	234 ± 57	193; 275	236 ± 56	195; 276	0.18	0.03	0.22

CMJ, countermovement jump; POWER, maximal 6 s power cycling; UPPER BODY STRENGTH, maximal isometric bilateral extension; LOWER BODY STRENGTH, maximal bilateral extension; TSG, task specific group; STG, strength training group; CON, control group. * = compared to PRE values * = *p* < 0.05, ** = *p* < 0.01; ^#^ = compared to MID values, ^#^ = *p* < 0.05, ^##^ *p* < 0.01.

**Table 3:** Simulated military task performance times for different tasks (first 5 m run, crawl, kettlebell carry (KB), casualty drag, and final 10 m run) from the first trial in the PRE, MID, and POST measurements points.

First Trial		PRE		MID		POST		|Effect Size|		
Performance Measure	Group	Mean (±SD)	95% CI	Mean (±SD)	95% CI	Mean (±SD)	95% CI	1 vs. 2	2 vs. 3	1 vs. 3
5 m run (s)	TSG	2.5 ± 0.3	2.4; 2.7	2.5 ± 0.4	2.3; 2.7	2.4 ± 0.3	2.3; 2.6	0.23	0.13	0.41
	STG	2.5 ± 0.2	2.4; 2.6	2.4 ± 0.2	2.3; 2.5	2.4 ± 0.2	2.3; 2.5	0.32	0.10	0.39
	CON	2.6 ± 0.4	2.3; 2.9	2.5 ± 0.3	2.3; 2.8	2.5 ± 0.3	2.3; 2.7	0.22	0.03	0.26
Crawl (s)	TSG	8.0 ± 2.0	7.0; 9.1	6.7 ± 1.8 ***	5.8; 7.7	6.4 ± 1.3 ***	5.7; 7.1	0.69	0.22	0.96
	STG	7.5 ± 1.4	6.8; 8.3	6.5 ± 1.2 **	5.8; 7.2	6.4 ± 1.7 **	5.4; 7.4	0.81	0.05	0.74
	CON	7.3 ± 1.0	6.5; 8.0	6.6 ± 1.8	5.4; 7.9	6.6 ± 1.8	5.3; 7.8	0.47	0.05	0.53
KB carry (s)	TSG	12.0 ± 1.9	11.0; 12.9	11.0 ± 1.7 ***	10.1; 11.8	11.1 ± 1.8 *	10.2; 12.0	0.58	0.08	0.50
	STG	11.5 ± 1.2	10.8; 12.1	10.8 ± 1.1 *	10.2; 11.4	10.9 ± 1.4	10.1; 11.7	0.61	0.07	0.47
	CON	11.5 ± 1.3	10.6; 12.4	11.2 ± 1.4	10.2; 12.2	11.2 ± 1.3	10.2; 12.1	0.22	0.04	0.26
Drag (s)	TSG	11.7 ± 3.7	9.8; 13.6	9.8 ± 2.4 ***	8.6; 11.1	9.7 ± 2.3 ***	8.5; 10.9	0.61	0.04	0.65
	STG	10.6 ± 2.4	9.3; 12.0	9.2 ± 1.6 **	8.4; 10.1	9.6 ± 1.9 *	8.5; 10.7	0.72	0.11	0.49
	CON	10.7 ± 2.0	9.3; 12.1	10.6 ± 3.2	8.3; 12.9	10.5 ± 2.9	8.4; 12.5	0.04	0.05	0.11
10 m run (s)	TSG	3.1 ± 0.4	2.9; 3.4	3.0 ± 0.4 *	2.8; 3.1	2.9 ± 0.3 *	2.8; 3.1	0.54	0.03	0.64
	STG	3.1 ± 0.4	2.9; 3.4	2.9 ± 0.3 ***	2.7; 3.0	2.9 ± 0.3 *	2.7; 3.0	0.74	0.18	0.72
	CON	3.1 ± 0.5	2.7; 3.4	3.0 ± 0.5	2.6; 3.4	3.0 ± 0.6	2.6; 3.4	0.14	0.04	0.10

TSG = soldier task specific group; STG = strength training group; CON = control group; * = compared to PRE values * = *p* < 0.05, ** = *p* < 0.01, *** = *p* < 0.001.

**Table 4:** Simulated military task performance times for different tasks (first 5 m run, crawl, kettlebell carry (KB), casualty drag, and final 10 m run) from the second trial in the PRE, MID, and POST measurement points.

Second Trial		PRE		MID		POST		|Effect Size|		
Performance Measure	Group	Mean (±SD)	95% CI	Mean (±SD)	95% CI	Mean (±SD)	95% CI	1 vs. 2	2 vs. 3	1 vs. 3
5 m run (s)	TSG	2.6 ± 0.4	2.4; 2.8	2.5 ± 0.3	2.3; 2.6	2.5 ± 0.3 *	2.3; 2.6	0.24	0.21	0.21
	STG	2.6 ± 0.2	2.4; 2.7	2.5 ± 0.2	2.4; 2.7	2.4 ± 0.2	2.3; 2.6	0.10	0.43	0.55
	CON	2.8 ± 0.2	2.7; 3.0	2.6 ± 0.5	2.3; 3.0	2.6 ± 0.4 *	2.4; 2.9	0.60	0.05	0.68
Crawl (s)	TSG	9.7 ± 2.9	8.2; 11.2	7.7 ± 2.0 ***	6.7; 8.7	7.1 ± 1.5 ***^,##^	6.3; 7.9	0.81	0.37	1.12
	STG	8.9 ± 1.9	7.8; 9.9	7.7 ± 1.5 *	6.9; 8.6	7.5 ± 1.9 ***	6.4; 8.5	0.69	0.16	0.77
	CON	8.4 ± 1.7	7.2; 9.7	7.8 ± 2.2	6.2; 9.4	7.1 ± 1.8 **^,#^	5.9; 8.4	0.33	0.35	0.78
KB carry (s)	TSG	14.1 ± 3.4	12,4; 15.8	12.3 ± 1.9 **	11.4; 13.3	11.9 ± 1.8 ***^,#^	11.0; 12.8	0.68	0.23	0.85
	STG	13.0 ± 1.5	12.2; 13.8	11.9 ± 1.1 ***	11.2; 12.5	11.6 ± 1.2 ***	10.9; 12.3	0.90	0.22	1.06
	CON	13.0 ± 2.0	11.6; 14.4	12.7 ± 2.1	11.2; 14.2	11.7 ± 1.6 **^,###^	10.6; 12.8	0.33	0.58	0.79
Drag (s)	TSG	14.2 ± 5.2	11.5; 16.9	11.5 ± 3.0 ***	9.9; 13.0	11.2 ± 2.8 ***	9.7; 12.6	0.66	0.10	0.74
	STG	13.6 ± 4.2	11.3; 15.9	10.9 ± 1.9 **	9.9; 12.0	10.8 ± 2.6 ***	9.3; 12.3	0.85	0.06	0.83
	CON	13.4 ± 2.9	11.3; 15.4	12.3 ± 3.8	9.7; 15.0	11.3 ± 3.5	8.8; 13.8	0.32	0.30	0.67
10 m run (s)	TSG	3.5 ± 0.6	3.2; 3.8	3.2 ± 0.4 *	3.0; 3.4	3.3 ± 0.4 *	3.1; 3.4	0.59	0.06	0.54
	STG	3.6 ± 0.4	3.3; 3.8	3.1 ± 0.3 ***	2.9; 3.3	3.1 ± 0.3 ***	3.0; 3.3	1.12	0.25	1.28
	CON	3.5 ± 4.0	3.2; 3.8	3.4 ± 0.7	2.8; 3.9	3.2 ± 0.5*	2.8; 3.5	0.30	0.26	0.78

TSG = soldier task specific group; STG = strength training group; CON = control group; * = compared to PRE values * = *p* < 0.05, ** = *p* < 0.01, *** = *p* < 0.001; ^#^ = compared to MID values ^#^ = *p* < 0.05, ^##^ = *p* < 0.01, ^###^ *p* < 0.001.

**Table 5:** Simulated military task performance times for different tasks (first 5 m run, crawl, kettlebell carry (KB), casualty drag, and final 10 m run) from the third trial in the PRE, MID, and POST measurement points.

Third Trial		PRE		MID		POST		|Effect Size|		
Performance Measure	Group	Mean (±SD)	95% CI	Mean (±SD)	95% CI	Mean (±SD)	95% CI	1 vs. 2	2 vs. 3	1 vs. 3
5 m run (s)	TSG	2.9 ± 0.6	2.6; 3.2	2.6 ± 0.3 ***	2.4; 2.8	2.6 ± 0.3 ***	2.4; 2.7	0.70	0.16	0.81
	STG	2.8 ± 0.3	2.6; 2.9	2.6 ± 0.2 **	2.5; 2.7	2.6 ± 0.3 *	2.4; 2.7	0.81	0.04	0.78
	CON	2.8 ± 0.5	2.5; 3.2	2.9 ± 0.7	2.5; 3.4	2.7 ± 0.5 ^##^	2.4; 3.0	0.22	0.43	0.25
Crawl (s)	TSG	11.2 ± 3.6	9.4; 13.1	8.9 ± 2.3 ***	7.7; 10.1	7.9 ± 1.8 ***^,##^	7.0; 8.9	0.80	0.47	1.19
	STG	10.6 ± 2.6	9.2; 12.1	8.5 ± 2.0 ***	7.4; 9.6	8.0 ± 1.6 ***	7.1; 8.8	0.93	0.30	1.26
	CON	9.0 ± 1.5	7.9; 10.1	9.2 ± 3.3	6.9; 11.6	7.4 ± 1.9 *^,#^	6.0; 8.8	0.08	0.71	0.99
KB carry (s)	TSG	15.4 ± 3.8	13.4; 17.3	13.4 ± 2.2 ***	12.2; 14.5	12.9 ± 2.7 ***	11.5; 14.3	0.67	0.20	0.79
	STG	14.0 ± 1.9	13.0; 15:0	12.6 ± 1.5 **	11.8; 13.4	11.9 ± 1.4 ***^,##^	11.1; 12.7	0.86	0.46	1.28
	CON	13.6 ± 1.7	12.4; 14.8	13.5 ± 2.4	11.8; 15.2	11.8 ± 1.9 **^,###^	10.4; 13.1	0.08	0.84	1.09
Drag (s)	TSG	15.9 ± 5.8	12.9; 18.9	13.0 ± 3.1 **	11.4; 14.6	11.9 ± 2.6 ***^,#^	10.6; 13.2	0.64	0.40	0.92
	STG	15.6 ± 5.6	12.5; 18.7	12.1 ± 3.4 ***	10.2; 14.0	11.9 ± 4.1 ***	9.6; 14.2	0.77	0.07	0.78
	CON	13.8 ± 2.9	11.7; 15.9	13.2 ± 3.8	10.4; 15.9	12.4 ± 3.3	10.0; 14.8	0.20	0.22	0.47
10 m run (s)	TSG	3.8 ± 0.6	3.5; 4.1	3.4 ± 0.3 **	3.2; 3.6	3.4 ± 0.4 *	3.2; 3.6	0.84	0.06	0.72
	STG	3.5 ± 0.3	3.3; 3.7	3.3 ± 0.5	3.0; 3.6	3.2 ± 0.4 *	3.0; 3.4	0.40	0.25	0.83
	CON	3.6 ± 0.5	3.2; 4.0	3.7 ± 0.7	3.1; 4.2	3.5 ± 0.8	2.9; 4.0	0.12	0.26	0.18

TSG = soldier task specific group; STG = strength training group; CON = control group; * = compared to PRE values * = *p* < 0.05, ** = *p* < 0.01, *** = *p* < 0.001; ^#^ = compared to MID values ^#^ = *p* < 0.05, ^##^ = *p* < 0.01, ^###^ *p* < 0.001.
